# Prospective relationships between patterns of religious belief/non-belief and mental health in adults: A UK cohort study

**DOI:** 10.1016/j.socscimed.2024.117342

**Published:** 2024-09-17

**Authors:** Isaac Halstead, Jon Heron, Connie Svob, Carol Joinson

**Affiliations:** a The Centre for Academic Child Health, Population Health Sciences, Bristol Medical School, University of Bristol, Bristol, BS8 2BN, UK; b The Centre for Academic Mental Health, Bristol Medical School, University of Bristol, Bristol, BS8 2BN, UK; c Department of Psychiatry, Vagelos College of Physicians and Surgeons, Department of Epidemiology, Mailman School of Public Health, Columbia University, Division of Child & Adolescent Psychiatry, New York State Psychiatric Institute, New York, NY, USA

**Keywords:** ALSPAC, Religion, Mental health, Latent class analysis, Religiosity

## 1 Introduction

Mental health problems in adulthood are linked to poor physical health (Mai et al., 2011) relationship quality (Pieh et al., 2020) and reduced quality of life (Chaves et al., 2014; Grill et al., 2020). Poor mental health can also be a source of shame or stigma (Al-Natour et al., 2021), creating an unhealthy feedback loop that can worsen symptoms (Clement et al., 2015).

There is evidence that a range of individual factors in adulthood are associated with poor mental health such as low socioeconomic position (SEP) (Muntaner, 2004), personality traits such as neuroticism (Bucher et al., 2019), substance use (Dagenhardt and Hall, 2001) and current life events (Cleland et al., 2016). Individual differences in religiosity are also thought to contribute to mental health outcomes in adulthood. Religiosity can be defined as a combination of beliefs, behaviours and rituals related to a higher or divine power (Koenig, 2009). Most previous research that has investigated associations between religiosity and mental health was conducted in the US and has found consistent evidence of an association between religiosity and better mental health (for a review, see Braam and Koenig, 2019; Koenig, 2009; Koenig et al., 2024). Estimates in 2009 suggest approximately 70% of research on religious belief and mental health is based on US samples (Koenig, 2009; Koenig et al., 2024). The US has a high prevalence of religious belief, which may be beneficial to those who are religious through having a majority social identity and access to a social network (Chatters et al., 2015; Merino, 2014). In a country with lower prevalence of religious belief, these benefits for religious individuals may not exist. This possibility has been suggested in the work of Gebauer et al. (2017); Stavrova (2015) who found evidence that religious individuals have better mental and physical health in countries with a religious culture.

Literature that has examined non-US samples found less consistent results. For example, (King et al., 2013) found no association between religiosity and anxiety, phobias, and depression in a UK sample. Furthermore, (Leurent et al., 2013) found a negative association between religiosity and mental health in a UK sample, and a high degree of heterogeneity in the relationship between religiosity and mental health in a multi-country sample. These findings suggest that more research needs to be conducted in non-US samples, to examine evidence of differences in the association between religiosity and mental health.

Different methods of measuring religiosity might explain differences between study findings. Most studies of religiosity rely upon one or two of the following measures e.g. religious affiliation, belief in the presence of a higher/divine power, attendance at a place of worship, and the importance of religion in the individual’s life (see Galek and Porter, 2010 for percentages of studies using each type of religious measure across a range of mental health). When used separately, these measures are unable to capture the variety of beliefs and behaviours associated with religion, which is a multidimensional construct (Bodling et al., 2013). Furthermore, different non-religious groups are likely to respond to these items in the same way, despite representing heterogenous groups. For example, when an Atheist or Agnostic individual is asked if they attend a place of worship (other than e.g. weddings and funerals), they are both likely to respond with *no*. This can lead to a study conflating these groups and missing differences between them (e.g. curvilinear relationships between religiosity and mental health where those who are highly religious or highly non-religious have a lower risk of mental health problems (Galen and Kloet, 2011; Gontijo et al., 2022; Henderson and Kent, 2021).

If we use methods that allow qualitatively different groups of religious (non)believers to emerge, we may see more nuance in our results. For example, using latent class analysis allows us to see heterogeneous patterns of response, rather than single items or scales that provide a continuum of response. This has been shown previously to provide nuanced differences in religious (non)belief (Bravo et al., 2016; Halstead et al., 2024; Pearce et al., 2013), such as the identification of spiritual but not religious individuals (Tong and Yang, 2018) and differential associations in several cognitive domains (Lindeman and Lipsanen, 2016).

Many studies of religion and mental health are cross-sectional, often with small, convenience samples (e.g. N less than 600 and frequent use of university students) (Smith et al., 2003). These limitations introduce selection bias, low statistical power, reverse causality (Maselko et al., 2012) and the possibility of any associations being at least partially due to confounding. However, a number of studies have examined religious belief and mental health longitudinally, with a recent meta-analysis finding a small but consistent association between religious belief and mental health (Garssen et al., 2021).There are also studies that combine longitudinal samples with explorations of non-linear associations between religion and mental health. These studies found, in US samples, that those who engage in high levels or low levels of religious behaviours (e.g. attendance at a place of worship) had better mental health than those who only engaged in moderate levels of religious behaviour (King et al., 2007; Ronneberg et al., 2016; Schnittker, 2001). While based on US samples, they show the utility of examining non-linear associations between religious belief and mental health.

The current study uses data from a large UK birth cohort to examine the prospective relationship between self-reported religiosity in young adults (age 27 years) and subsequent mental health (assessed at ages 28 and 31). Our aims are to characterise different patterns of religiosity using latent class analysis and to examine the associations of the different classes with later mental health outcomes including depression, anxiety, self-harm, and mental wellbeing.

## 2 Methods

### 2.1 Participants

The Avon Longitudinal Study of Parents and Children (ALSPAC) was established to understand how genetic and environmental characteristics influence health and development in parents and children. All pregnant women resident in a defined area in the Southwest of England, with an expected date of delivery between April 1, 1991 and December 31, 1992 were invited to take part in the study. The initial number of pregnancies enrolled is 14,541. Of these initial pregnancies, there was a total of 14,676 foetuses, resulting in 14,062 live births and 13,988 offspring who were alive at 1 year of age. These parents and offspring have been followed over the last 30 years and have completed a variety of questionnaires concerning their demographics, physiological and genetic data, life events, physical, and psychological characteristics. For more information, see (Boyd et al., 2013; Fraser et al., 2013; Northstone et al., 2019). The study website contains details of all data that is available through a fully searchable data dictionary and variable search tool (http://www.bristol.ac.uk/alspac/researchers/our-data/). Study data were collected and managed using REDCap electronic data capture tools hosted at the University of Bristol (Harris et al.,2009). REDCap (Research Electronic Data Capture) is a secure, web-based software platform designed to support data capture for research studies.

We conducted the primary analysis on an imputed dataset of 4165 participants who had maternal religiosity measures (see Iles-Caven et al., 2021 for descriptive statistics of the religiosity items). For full details of our imputation methodology, see the supplementary materials. We also conducted the analysis on the sample with complete data (585 participants with complete religiosity, mental health outcomes and confounders). We report these results in the supplementary materials.

**Fig. 1:**
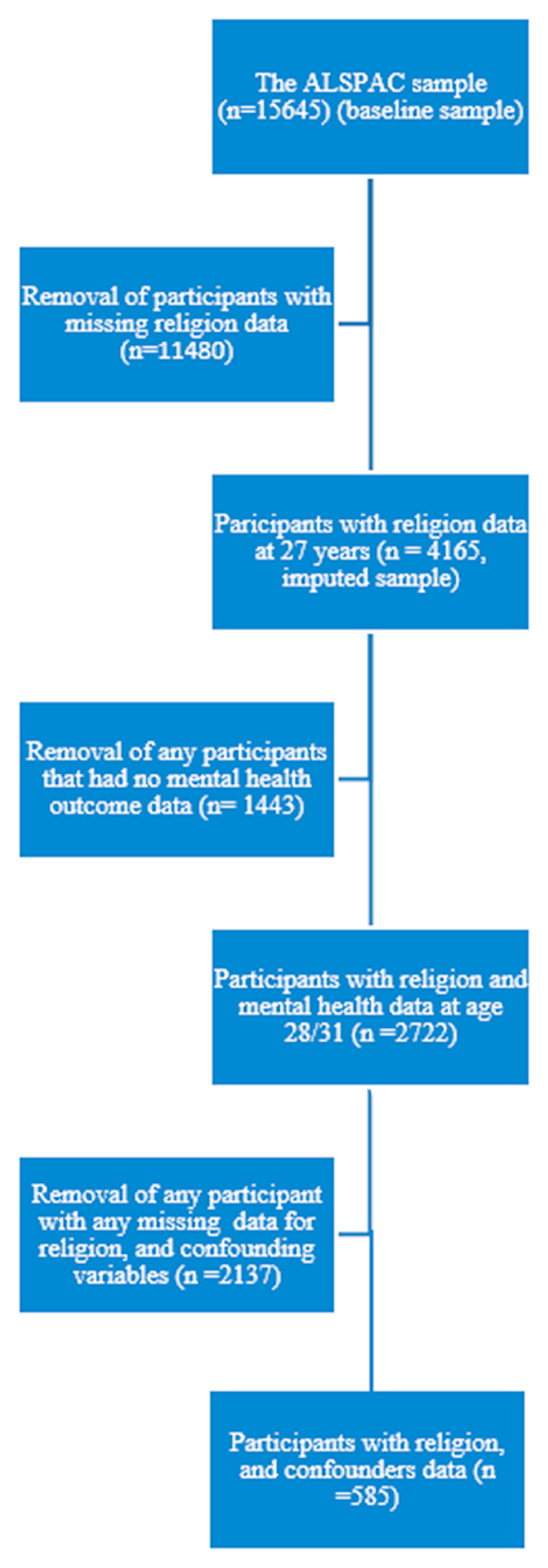
Flowchart for derivation of study sample.

Compared to the complete case sample, the imputed sample had a higher proportion of indicators of lower SEP (e.g. non-managerial/professional social class, fewer years in education, lower income), as well as higher proportions of all mental health outcomes. See Fig. 1 for details of and Table 1 for details of the complete case and imputed sample.

### 2.2 Exposures: self-reported items on religious beliefs and behaviour at age 27 years

We chose a range of items intended to capture both beliefs and behaviours associated with religiosity, as well as items that allowed us to differentiate between degree of religious belief, and types of non-belief. For more information regarding the questions used, see Table 2.

Latent class analysis is a ‘person-centred’ statistical technique (Nylund et al., 2007) that uses a set of observed variables and the conditional probability of responding in a particular pattern to those variables to probabilistically assign participants to a mutually exclusive unobserved group (i.e. latent class). We used data on religiosity assessed when the participants were 27 years old, because these were the most proximal variables to the adult mental health outcomes.

### 3.1 Outcomes: mental health at age 28–31 years

#### 3.1.1 Depression measured at 31

The Short Mood and Feelings Questionnaire (SMFQ) (Angold and Costello, 2013) is a brief (13-item) questionnaire that asks about the occurrence of depressive symptoms over the past 2 weeks. Scores equal to or greater than 11 are used to indicate high levels of depressive symptoms (Eyre et al., 2021). We also included the Edinburgh Postnatal Depression Scale (EPDS), which asks about the occurrence of depressive symptoms over the past week. Scores greater than 13 are used to identify the possible presence of depression (Levis et al., 2020). The EPDS has also been validated in use in men, and outside of the antenatal period (Matthey et al., 2001; Pearson et al., 2013).

#### 3.1.2 Anxiety measured at 28

The Generalised Anxiety Disorder Assessment (GAD-7) (Spitzer et al., 2006) is a brief 7-item questionnaire that asks about the occurrence of anxiety symptoms over the past 2 weeks. Scores higher than 7 were used as the cut off for the presence of anxiety (Plummer et al., 2016).

#### 3.1.3 Self-harm measured at 28

Participants were asked to indicate whether they had ever tried to, or thought about, harming or killing themselves in the past year. This measure is approximately equivalent to using hospital admission data, although there can be a small inconsistency between the two (Mars et al., 2016).

#### 3.1.4 Mental wellbeing measured at 28

The Warwick-Edinburgh Mental wellbeing Scales (WEMWBS) (Tennant et al., 2007) is a 14-item measure that asks questions concerning aspects of an individual’s mental wellbeing. Scores lower than 43 were used as a cutoff for low mental wellbeing (Kwong et al., 2021).

### 3.2 Confounders

Analyses were adjusted for factors that could potentially have a causal role in both religiosity and mental health, such as; socioeconomic factors (self-reported income, educational attainment, occupational social class), maternal religiosity (as measured by belief in God and attendance at a place of worship) (Li et al., 2016; Maselko et al., 2012), aspects of parenting in adolescence (parent and offspring reported time spent together and parental monitoring) (Cadman et al., 2022; Kim-Spoon et al., 2014), stressful life events (McLaughlin and Hatzenbuehler, 2009; Stauner et al., 2019), participant sex at birth, and mental health/well-being prior to the assessment of religiosity (depression, anxiety, depression, self-harm and wellbeing at age 25). For more information in these variables see the supplementary materials.

### 3.3 Statistical analyses

#### 3.1.1 Extraction of the religiosity latent classes

We used a 4-class solution for the religiosity latent classes, with measurement model parameters (class-specific item thresholds) taken from previous religiosity latent classes that were extracted from data from parents in the ALSPAC cohort (generated in Halstead et al., 2024) the original 6 items used from the previous study in conjunction with 7 additional religiosity items (see Table S1 of the supplemental materials) that were completed by the participants at age 27. The item thresholds for these additional items were freely estimated. We used this method to retain a 4-class solution, which shared measurement properties, and hence interpretation, with the earlier parental religiosity model, while providing more information about the classes through the inclusion of new items. See the results section for class shares for each latent class.

#### 3.3.2 Estimation of associations between latent classes and mental health outcomes

We used multivariable logistic regression to calculate odds ratios and 95% confidence intervals for the association between the religious latent classes and each mental health outcome. Odds ratios are presented in reference to the atheist class, which was the largest class (64%). Parameter estimates were then adjusted for confounders. The dataset was constructed in R studio (R Core Team, 2021) and all analyses were carried out in Mplus (Version 8.7), using a bias adjusted 3-step latent class analysis which incorporates uncertainty in latent class assignment (Vermunt and Magidson, 2021).

## 4 Results

We extracted latent classes that described different patterns of religiosity and we labelled the classes atheist, agnostic, moderately religious, and highly religious. The atheist class (≈64% of the sample) is typified by their consistent negative responses to all items. The agnostic class (≈19%) express a general uncertainty about the existence of a divine power, are unlikely to engage in any religious activities, but a small proportion of them report being slightly spiritual. The moderately religious class (≈13%) believe in a divine power, hold some religious beliefs, but are unlikely to attend church, or identify as being strongly religious. The highly religious class (≈4%) respond positively to all religiosity items. For more information regarding the conditional probabilities for each class, see Table 3.

Table 4 shows the results of the logistic regression analysis of the association between religiosity latent classes and mental health outcomes After adjusting for maternal religiosity, parent and offspring reported time spent together and parental monitoring, stressful life events, participant sex at birth, and mental health/well-being prior to the assessment of religiosity, the agnostic class, compared to the atheist class, had increased odds of high levels of depressive (SMFQ) and anxiety symptoms (GAD-7). In the unadjusted model, there was evidence of increased odds of depression (EPDS) in the agnostic class, compared with atheist class, but this association was attenuated in the fully adjusted model. There was weaker evidence that, compared with the atheist class, the moderately religious class had increased odds of depression and anxiety, and the highly religious class had reduced odds of low mental wellbeing and depressive symptoms.

There was little evidence of associations between the religiosity latent classes and mental health/well-being in the analysis with the complete case sample, with the exception of increased odds of anxiety in the moderately religious, compared with the atheist class. See Table S3 for details of the complete case analyses.

## 5 Discussion

Compared to the Atheist class, membership of the agnostic class was associated with higher odds of SMFQ depressive symptoms and GAD-7 anxiety symptoms. These associations remained after adjusting for confounders. There was also weak evidence that membership to the moderately religious class, compared with atheist class, was associated with higher odds of EPDS depression, and the highly religious class, compared with atheist class, had reduced odds of low mental wellbeing and depressive symptoms. There was a lower proportion of participants assigned to the moderately and highly religious classes, which resulted in less precision in the estimated associations.

### 5.1 Comparisons with previous research

Our findings relating to the highly religious class contrast with research, based on US samples, which mostly finds that religiosity is associated with better mental health. When comparing our findings to US samples, our work more closely aligns with the work of (Galen and Kloet, 2011), who found evidence for a curvilinear relationship between religiosity and mental health. Specifically, those with greater belief or non-belief had better mental health, whereas those with more moderate/uncertain beliefs had poorer mental health outcomes. This finding has been replicated in other studies (Gontijo et al., 2022; Henderson and Kent, 2021), suggesting that a linear association may be an inadequate explanation for the relationship between religiosity and mental health. It has been suggested that having confidence in a consistent worldview prevents ideological doubt and subsequently reduces psychological distress (Krause et al., 1999). This would account for the higher odds of mental health issues in the agnostic class, and tentative evidence for the same in the moderately religious class in the present study.

Another possible explanation for the findings in the agnostic class could be due to the probability of members of that class holding a small amount of spiritual belief. In the UK and other non-US samples, those who are spiritual are more likely to be depressed or anxious (King et al., 2013; Leurent et al., 2013), and this association is found in those with spiritual beliefs, but an absence of religiosity (King et al., 2006). The current findings contrast with our previous work that examined associations between maternal religiosity and childhood/adolescent mental health in the ALSPAC cohort. We previously found that offspring of agnostic mothers had better mental health outcomes, and offspring of highly religious/atheist mothers had poorer mental health outcomes in depression and anxiety (Halstead et al., 2023, 2024). This may reflect generational differences in the role or importance of (non)religiosity, both in the individual and society. The UK has become more secular over time (Major-Smith et al., 2023; Wilkins-Laflamme, 2014). Many of those identifying previously as agnostic or moderately religious have become atheist (while the highly religious class remains small but relatively stable in the last 3 decades within ALSPAC) (Major-Smith et al., 2023). Due to this change in religious belief over time, it may be more beneficial to be an atheist or highly religious in the contemporary UK due to being a member of a majority social identity, or a resilient and close-knit, but small, social group.

### 5.2 Possible mechanisms

As mentioned previously, the work of (Galen and Kloet, 2011) suggests that there are benefits to having a clear and coherent worldview, whether that be within a religious or non-religious framework. In a similar vein, being generally uncertain about existential issues such as the presence of a higher power is associated with a lower sense of purpose or meaning in life (Gontijo et al., 2022), which may in turn negatively impact their mental health (Boreham and Schutte, 2023). Additionally, the UK has become more (non)religiously polarised (Major-Smith et al., 2023; Wilkins-Laflamme, 2014). With fewer individuals occupying the middle ground, being Agnostic may now represent an outgroup, and the downsides associated with being a member of an outgroup, such as worse mental health (McIntyre et al., 2018). However, these possibilities have yet to be explored longitudinally, or with attention paid to causality, through methods such as propensity score matching (Austin, 2011).

### 5.3 Strengths and limitations

A high proportion of previous research that has examined the association between religiosity and mental health/well-being is based on US samples. This study, based on data from a large contemporary community-based cohort in the UK, allowed us to examine if these associations differ in a country with different proportions of religious individuals in the population (Duffy et al., 2023), which may also result in different confounding structures existing in these two countries (Santos et al., 2019). Major strengths include the prospective study design, the use of validated self-report questionnaires for assessing mental health and well-being, the examination of different patterns (latent classes) of religious belief/non-belief compared with previous studies which tend to use single item measures of religiosity, and adjustment for a wide range of empirically informed confounders. The use of latent classes provided insights into patterns of beliefs/behaviours (e.g. if they would appeal to god, or how spiritual they consider themselves to be) that may have been obscured by only using items such as place of worship attendance, that fail to differentiate between Agnostic and Atheist individuals,both of which would be likely to indicate they do not attend a place of worship.

There are also limitations that should be considered when interpreting our findings. There was a large amount of attrition between the baseline sample, and the final complete case sample, which led to a loss of statistical power and potential selection bias. Specifically, those with higher SEP (Howe et al., 2013) and higher religiosity (Morgan et al., 2022) are more likely to participate in ALSPAC initially and continue to participate over time. As recommended by (Sterne et al., 2009), we report the results from the imputed data as the main findings and compare these with the results from the complete case analysis. There is evidence that multiple imputation eliminates bias regardless of the proportion of missing data (even with >50% missing data) (Madley-Dowd et al., 2019). Our study is limited in its generalisability, given the very small numbers of non-White, and non-Christian individuals, which prevents us from generalising to these groups.

### 5.4 Conclusions

Future work would benefit from replicating our analyses in non-UK samples to examine if the association between religiosity and mental health/well-being differs. Repeating our analysis in samples with other religious affiliations would also be beneficial to advance understanding of possible heterogeneity in associations according to religious affiliation. Finally, the mechanisms behind the association between religiosity and mental health need to be explored, such as meaning or purpose in life.

## Supplementary Material

Supplementary Materials

## Figures and Tables

**Table 1:** Distribution of variables across the complete case and imputed datasets for demographic and mental health outcomes.

		Complete case sample (585)	Imputed Sample (4165)
Assigned sex at birth (Ref: Female)	Male	33.9%	34.3%
Social class (Ref: managerial/professional)	Non-managerial/professional	49.8%	54.8%
Parental social class (Ref: non-manual)	Manual	5.7%	8.8%
Years in education	Median	16	15
Total take home pay after tax each month	Median	4 (£1500 - £1999)	3 (£1000 - £1499)
Stressful life events	Median	2	2
High levels of depressive symptoms (Ref: none)	Yes	17.3%	23.8%
EPDS depression (Ref: none)	Yes	20.7%	26.1%
GAD 7 anxiety (Ref: none)	Yes	21.5%	25.3%
Self-harm (Ref: no)	Yes	5.1%	5.9%
Self-harm thoughts (Ref: no)	Yes	19.2%	20.0%
Mental wellbeing (Ref: normal/high mental wellbeing)	Low mental wellbeing	23.1%	27.2%

Note: the numbers for the income variable refer to the following categories 1 £1 - £499, 2 £500 - £999, 3 £1000 - £1499, 4 £1500 - £1999, 5 £2000 - £2499, 6 £2500 - £299, 7 £3000 and above.

**Table 2:** Religion items used and response options.

Variable name	Description	Response options
YPG3000	Belief in God or some divine power	Yes
		Not sure
		No
YPG3010	Feel that God (or some divine power) has helped them at any time	Yes
		Not sure
		No
YPG3020	Would appeal to God (or some divine power) for help if in trouble	Yes
		Not sure
		No
YPG3030	Prays even if not in trouble	Yes
		Not sure
		No
YPG3050	Length of time had this faith/belief	Less than 5 years
		More than 5 years
YPG3080	Frequency attend church/temple/mosque/other religious meetings	Regular
		Occasional
YPG3090-93	Obtain help/support from: any member of a religious group	Yes
		No
YPG3130	In my life, I experience the Presence of the Divine (e.g. God)	Definitely true of me/Tends to be true of me
		Unsure
		Tends not to be true of me/Definitely not true of me
		Not applicable
YPG3140	Religious beliefs lie behind whole approach to life	Definitely true of me/Tends to be true of me
		Unsure
		Tends not to be true of me/Definitely not true of me
		Not applicable
YPG3160	Attends place of worship because it helps them to make friends	Strongly agree/Mildly agree
		Not sure
		Mildly disagree/Strongly disagree
		Not applicable
YPG3170	Prays mainly to gain relief and protection	Strongly agree/Mildly agree
		Not sure
		Mildly disagree/Strongly disagree
		Not applicable
YPG3210	Extent respondent considers themselves a religious person	Very religious
		Moderately religious
		Slightly religious
		Not religious at all
YPG3220	Extent respondent considers themselves a spiritual person	Very spiritual
		Moderately spiritual
		Slightly spiritual
		Not religious at all

**Table 3:** Conditional probabilities and class shares for the latent classes.

Class share:	Atheist .64	Agnostic .19	Moderately religious .13	Highly religious .04
**Belief in god/divine power**				
Yes	.05	.22	.93	.99
Not sure	.25	.77	.07	.01
No	.70	.01	.00	.00
**Pray when in trouble**				
Yes	.01	.07	.44	1.00
Not sure	.01	.19	.19	.01
No	.99	.74	.37	.00
**Attendance**				
Regular	.01	.07	.17	.96
Occasional	.99	.93	.83	.04
**Extent they consider themselves religious**				
Very/moderately	.00	.01	.20	.75
Slightly	.02	.34	.61	.16
Not at all	.98	.66	.19	.10
**Extent they consider themselves spiritual**				
Very/moderately	.05	.17	.39	.89
Slightly	.21	.42	.40	.10
Not at all	.74	.41	.21	.01
**Has God helped them**				
Yes	.00	.02	.69	.94
Not sure	.02	.77	.28	.06
No	.98	.21	.03	.00
**Would they appeal to God**				
Yes	.02	.23	.88	.98
Not sure	.13	.68	.10	.01
No	.86	.10	.03	.00
**Duration of belief**				
More than 5 years	.98	1.00	.97	.92
Less than 5 years	.02	.00	.03	.08
**Help from religious individuals**				
Yes	.00	.02	.04	.84
No	1.00	.98	.96	.17
**Feels the presence of divine**				
Definitely/tends to be true of me	.00	.01	.32	.97
Unsure	.01	.23	.40	.03
Definitely/tends not to be true of me	.01	.19	.24	.00
Not applicable	.98	.57	.03	.00
**Religion lies behind approach to life**				
Definitely/tends to be true of me	.02	.04	.23	.92
Unsure	.02	.09	.27	.06
Definitely/tends not to be true of me	.01	.12	.45	.02
Not applicable	.95	.75	.06	.00
**Attends church to make friends**				
Definitely/tends to be true of me	.00	.02	.08	.63
Unsure	.00	.02	.06	.02
Definitely/tends not to be true of me	.00	.00	.39	.28
Not applicable	.99	.96	.48	.07
**Prays for relief and protection**				
Definitely/tends to be true of me	.00	.08	.41	.52
Unsure	.00	.07	.17	.09
Definitely/tends not to be true of me	.00	.02	.25	.38
Not applicable	1.00	.83	.18	.02

**Table 4:** Odds ratios and 95% confidence intervals for the association between the religiosity latent classes and mental health outcomes (n = 4165).

		Atheist (Ref)	Agnostic	Moderately religious	Highly religious	Omnibus p-value
High levels of SMFQ depressive symptoms	Unadjusted	1.0	1.53 (1.24,1.90)	1.24 (0.97,1.59)	0.71 (0.44,1.14)	<0.001
	Adjusted	1.0	1.55 (1.11,2.16)	1.04 (0.75,1.45)	0.59 (0.35,1.01)	0.007
EPDS depression	Unadjusted	1.0	1.25 (1.01,1.54)	1.41 (1.12,1.78)	1.03 (0.69,1.54)	0.016
	Adjusted	1.0	1.12 (0.82,1.52)	1.27 (0.94,1.71)	0.97 (0.61,1.55)	0.443
GAD-7 anxiety	Unadjusted	1.0	1.41 (1.14,1.74)	1.18 (0.93,1.50)	0.92 (0.61,1.39)	0.010
	Adjusted	1.0	1.39 (1.03,1.88)	0.94 (0.69,1.28)	0.82 (0.51,1.33)	0.104
Self-harm actions	Unadjusted	1.0	1.11 (0.74,1.66)	1.17 (0.76,1.78)	1.32 (0.66,2.64)	0.771
	Adjusted	1.0	1.07 (0.60,1.90)	0.97 (0.56,1.68)	1.27 (0.57,2.86)	0.946
Self-harm thoughts	Unadjusted	1.0	1.17 (0.93,1.47)	1.12 (0.87,1.44)	0.94 (0.60,1.47)	0.467
	Adjusted	1.0	1.17 (0.83,1.64)	1.06 (0.76,1.48)	0.86 (0.51,1.45)	0.709
Low WEMWBS mental wellbeing	Unadjusted	1.0	1.11 (0.90,1.37)	1.04 (0.82,1.31)	0.64 (0.42,0.99)	0.142
	Adjusted	1.0	1.09 (0.81,1.47)	1.03 (0.77,1.37)	0.67 (0.41,1.08)	0.350

**Note**. The fully adjusted model adjusts for socioeconomic factors, maternal religiosity, parent and offspring reported time spent together and parental monitoring, stressful life events, participant sex at birth, and mental health/well-being prior to the assessment of religiosity. P values are omnibus p-values based on a Wald test with 3 d.f.

## Data Availability

Please see the ALSPAC data management plan which describes the policy regarding data sharing (http://www.bristol.ac.uk/alspac/researchers/data-access/documents/alspac-data-management-plan.pdf), which is by a system of managed open access. Data used for this submission will be made available on request to the Executive (alspac-exec@bristol.ac.uk). The datasets presented in this article are linked to ALSPAC project number B4335, please quote this project number during your application. The steps below highlight how to apply for access to the data included in this study and all other ALSPAC data. Please read the ALSPAC access policy (http://www.bristol.ac.uk/media-library/sites/alspac/documents/researchers/data-access/ALSPAC_Access_Policy.pdf) which describes the process of accessing the data and samples in detail, and outlines the costs associated with doing so. You may also find it useful to browse our fully searchable research proposals database (https://proposals.epi.bristol.ac.uk/?q=proposalSummaries), which lists all research projects that have been approved since April 2011. Please submit your research proposal (https://proposals.epi.bristol.ac.uk/) for consideration by the ALSPAC Executive Committee. You will receive a response within 10 working days to advise you whether your proposal has been approved.

## Data availability

I have included the ALSPAC study’s data access policy in the manuscript.
