# Supplementary material for: Prospective relationships between patterns of religious belief/non-belief and mental health in adults: A UK cohort study
Source: Soc Sci Med. Author manuscript; Available in PMC 2024 Dec 9. (PMC7617112; doi:10.1016/j.socscimed.2024.117342)
Supplement: Supplementary Materials [file EMS200189-supplement-Supplementary_Materials.docx]

**Table S1.** Variable information.

| Construct | Scale | Variable name | Question | Time point | Recoding |
| --- | --- | --- | --- | --- | --- |
| **Exposure variables** |  |  |  |  |  |
| Religious belief |  | YPG3000  YPG3010  YPG3020  YPG3030  YPG3050  YPG3080  YPG3090-93  YPG3130  YPG3140  YPG3160  YPG3170  YPG3210  YPG3220 | - Belief in God or some divine power - Feel that God (or some divine power) has helped them at any time - Would appeal to God (or some divine power) for help if in trouble - Prays even if not in trouble - Length of time had this faith/belief - Frequency attend church/temple/mosque/other religious meetings - Obtain help/support from: leaders of own religious group - Obtain help/support from: Other members of own religious group - Obtain help/support from: Leaders of other religious groups - Obtain help/support from: Members of other religious groups Experiences the Prescence of the Divine (e.g., God) in their life - Religious beliefs lie behind whole approach to life - Experiences the Prescence of the Divine (e.g., God) in their life - Attends place of worship because it helps them to make friends - Prays mainly to gain relief and protection - Extent respondent considers themselves a religious person - Extent respondent considers themselves a spiritual person | 27 Years old | Participants that indicated they did not believe in a divine power or were unsure and responded to items with strongly disagree, were recoded to not applicable due to them possibly using a strongly disagree response as a not applicable response.    Participants who had received any help from any religious group were combined into a single measure of help |
| **Outcome variables** |  |  |  |  |  |
| Depression (Outcome) | SMFQ | covid4yp_4066 | - Total score - Moods and feelings Q: Dichotomised at 12: COVID4 | 30 Years old | Scores equal to or greater than 11 were coded as 1 (Kwong et al., 2021). |
| Depression2 (Outcome) | EPDS | covid5yp_4010 | - Total score - EPDS: COVID5 | 31 Years old | Scores equal to or greater than 13 were coded as 1 (Paul and Pearson., 2020). |
| Self-harm acts (Outcome) |  | YPH6010  YPH6020 | - YP has ever hurt themselves on purpose (e.g. by taking an overdose of pills) - Frequency YP has hurt themselves on purpose in the past year | 29 Years old | The presence of self-harm in the past year was coded as 1 |
| Self-harm thoughts (Outcome) |  | YPH6050  YPH6060 | - YP has thought of killing themselves, even if they would not actually do it - Last time YP thought of killing themselves, even if they would not do it | 29 Years old | The presence of any self-harm thoughts in the past year was coded as 1. |
| Anxiety (Outcome) | GAD7 | covid4yp_4081 | - Total score - Gad7: Categories of anxiety: COVID4 | 30 Years old | Scores higher than 10 were coded as 1 (Kwong et al., 2021). |
| Wellbeing (Outcome | WEMWBS | covid4yp_4120 | - Total score - Warwick Edinburgh Mental wellbeing Scale: COVID4 | 30 Years old | Scores equal to or less than 40 were coded as 1 (Shakeshaft et al., 2023). |
| **Confounders** |  |  |  |  |  |
| Wellbeing (confounder) | WEMWBS | YPC0600 | - WEMWBS Composite | 23 Years old | Scores equal to or less than 40 were coded as 1 (Shakeshaft et al., 2023). |
| Anxiety (Confounder) | SCAARED | YPE2000  YPE2001  YPE2002  YPE2003  YPE2004  YPE2005  YPE2006  YPE2007  YPE2008  YPE2009  YPE2010  YPE2011  YPE2012  YPE2013  YPE2014  YPE2015  YPE2016  YPE2017  YPE2018  YPE2019  YPE2020  YPE2021  YPE2022  YPE2023  YPE2024  YPE2025  YPE2026  YPE2027  YPE2028  YPE2029  YPE2030  YPE2031  YPE2032  YPE2033  YPE2034  YPE2035  YPE2036  YPE2037  YPE2038  YPE2039  YPE2040  YPE2041  YPE2042  YPE2043 |  | 25 Years old | A sum of these items was created, and scores equal to or greater than 23 were coded as 1 (Angulo et al., 2017) |
|  |  |  |  |  |  |
| Depression (Confounder) | SMFQ | YPE4080  YPE4082  YPE4083  YPE4084  YPE4085  YPE4086  YPE4088  YPE4089  YPE4091  YPE4092  YPE4093  YPE4094  YPE4095 |  | 25 Years old | Scores equal to or greater than 11 were coded as 1 (Kwong et al., 2021). |
| Self-harm acts (Confounder) |  | YPE4040  YPE4030 | - Over the whole of YP's lifetime, YP has tried to harm/kill themselves - YP tried to harm/kill themselves when YP was sad/lacking interest | 25 Years old | The presence of any lifetime self-harm was coded as 1. |
| Self-harm thoughts (Confounder) |  | YPD6040 | - YP ever thought of killing themself, even if would not really do it | 24 Years old | The presence of any self-harm thoughts was coded as 1. |
| Own income |  | YPE6020 | - YP's total take-home pay each month after tax & national insurance removed | 25 Years old | 0 Not doing paid work  1 £1 - £499  2 £500 - £999  3 £1000 - £1499  4 £1500 - £1999  5 £2000 - £2499  6 £2500 - £2999  7 £3000 and above |
| Own education |  | YPF7510  YPF7980 | - YP has no educational qualifications - Years of education attained | 26 Years old |  |
| Parental occupational social class |  | C755/C765 | - Social Class – Maternal - Social Class - Paternal | Antenatal | The highest social class between each parent was used. |
| Own social class |  | YPC2492 | - NS-SEC Occupational class (5 categories) | 23 Years old | This was recoded into managerial/administrative/professional and non-managerial/administrative/professional |
| Parental RSBB |  | p4040  p4049 | - Mother believes in God or some divine power - Mother goes to a place of worship | 9 Years old | Yes, not sure, no    Yes, at least once a week yes, at least once a month yes, at least once a year not at all |
| Mothers parenting score/Offspring parenting score |  | fh8220  fh8221  tc1000  tc1001  fh8200  fh8201  fh8202  fh8203  fh9813  fh9814  fh9818  fh9819  fh8230  fh8231  fh8232  fh8233  fh8234  fh8235 | - Number of hours YP spends doing things with parents, on weekdays - Number of hours YP spends doing things with parents, on weekends - Frequency respondent usually spend having a conversation with study teenager - Frequency respondent's husband/partner/someone else usually spend having a conversation with study teenager - Frequency parents knew where YP was going, when YP went out, in last year - Frequency parents knew who YP was going out with, when YP went out, in last year - Frequency parents knew what YP was doing, when YP went out, in last year - Frequency parents knew what time YP would be home, when YP went out, in last year - Frequency carer knows what YP does in their free time - Frequency carer knows what YP spends their money on - Frequency YP tells carer what they did/where they were in the evening   Frequency carer asks YP what they did in their free time | 15.5 Years old | Individual sum scores were generated for offspring reported parenting quality, parent reported parenting quality, offspring reported parental monitoring, and parent reported parental monitoring. |
| Stressful life events score |  | YPE6520  YPE6530  YPE6590  YPE6600  YPE6610  YPE6630  YPE6640  YPE6650  YPE6660  YPE6670  YPE6680  YPE6690  YPE6700  YPE6710  YPE6730  YPE6740 | - YP/partner became pregnant in past 12 months - YP/partner had a baby in past 12 months - YP got divorced/separated in past 12 months - YP admitted to hospital in past 12 months - YP in trouble with the law in past 12 months - YP's house/car burgled/stolen past 12 months - YP's pet died in past 12 months - YP's parent died in past 12 months - YP's friend died in past 12 months - YP's/partner's child died in past 12 months - YP/partner had miscarriage in past 12 months - YP's relative(not parent) died past 12 months - YP became homeless in past 12 months - YP had big financial problems past 12 months - YP/partner had abortion past 12 months - YP's parents divorced/separated past 12 months | 26 Years old | A sum score was calculated for these items based on whether the event had occurred and how much it had affected them |
| **Auxiliary variables** |  |  |  |  |  |
| Common mental disorders | CIS-R | FJCI050 | - Score: Total cis-r score | 18 Years old |  |
| Depression | SMFQ | CCXD917 | - Moods and Feelings total score | 18 Years old |  |
| Depression | ICD 10 classification (CIS-R) | FJCI1001 | - Participant has ICD-10 diagnosis of depression: TF4 | 18 Years old |  |
| Anxiety | GAD 7 | FJCI602 | - Presence of gad symptoms: TF4 | 18 Years old |  |
| Self-harm |  | FJCI369  FJCI370 | - Self-harm indicator: TF4 - Number of self-harm incidents in last year: TF4 | 18 Years old |  |
| Self-harm thoughts |  | FJCI371 | - Thoughts of self-harm in past week: TF4 | 18 Years old |  |
| Depression | SMFQ | YPA2000  YPA2010  YPA2020  YPA2030  YPA2040  YPA2050  YPA2060  YPA2070  YPA2080  YPA2090  YPA2100  YPA2110  YPA2120 |  | 21 Years old | Scores greater than or equal to 11 were coded as 1 (Kwong et al., 2021). |
| Anxiety | GAD7 | YPA2160  YPA2170  YPA2180  YPA2190  YPA2200  YPA2210  YPA2220 |  | 21 Years old | Scores higher than 10 were coded as 1 (Kwong et al., 2021) |
| Parent housing status |  | a006 |  | Antenatal |  |
| Age of mother at birth |  | e695 |  | Antenatal |  |
| Parental education |  | c645a |  | 5 Years old  Antenatal |  |
| Parental mental health |  | T3255  pl6100 |  | 18 Years old  8 Years old |  |
| Smoking during pregnancy |  | e170 |  | Antenatal |  |
| Financial difficulties/problems |  | h735, c525,  b594 |  | 3 Years old  Antenatal  Antenatal |  |
| Antisocial behaviour in adolescence |  | fh8300  fh8301  fh8302  fh8303  fh8304  fh8305  fh8306  fh8307  fh8308  fh8309  fh8310  fh8311  fh8312  fh8313  fh8314  fh8315  fh8316  fh8317  fh8318  fh8319  fh8320  fh8321 |  | 16 years old |  |

*Anxiety at 25 years*

The Screen for Child Anxiety Related Disorders (SCAARED) (Birmaher et al., 1999) is a 41-item questionnaire that asks about the presence of anxiety symptoms. Scores higher than 25 were used as the cut off for the presence of anxiety (Angulo et al., 2017) and dichotomised.

*Depression at 25 years*

The Short Mood and Feelings Questionnaire (SMFQ) (Angold & Costello, 2013) is a brief (13-item) questionnaire that asks about the occurrence of depressive symptoms over the past 2 weeks. Scores equal to or greater than 11 were used as the cut off for the presence of depression (Thapar & McGuffin, 1998) and has been validated for use in adulthood (Eyre et al., 2021) and dichotomised.

*Self-harm*

Participants were asked to respond to indicate whether they had ever tried to, or thought about, harming or killing themselves over their whole lifetime. Measured at 25.

*Wellbeing at 23 years*

The Warwick-Edinburgh Mental Wellbeing Scales (WEMWBS) (Tennant et al., 2007) is a 14-item measure that asks questions about an individual’s wellbeing over the past 2 weeks. Scores lower than 43 were used as a cutoff for low wellbeing (Kwong et al., 2021) and dichotomised.

See Supplementary Table S1 for more information.

***Multiple imputation***

We used multiple imputation by chained equations to impute missing data on the mental health outcomes and confounders using the *mice* package in R (Buuren & Groothuis-Oudshoorn, 2011) under the Missing at Random (MAR) assumption. In addition to variables in the main analysis, we included auxiliary variables that were likely to be related to the missing data in the imputation model. These included earlier measures of depression, anxiety, and self-harm at age 8, 18, and 21, adolescent antisocial behaviours (16 years old), as well as parental mental health measures (8 years old ,18 years old), home ownership (antenatal), smoking during pregnancy (antenatal), age of mother at birth (antenatal), parental education (5 years old, antenatal), and parental financial difficulties (3 years old, antenatal) We used *mice to* impute 100 datasets with 100 iterations per dataset and the resulting text files were used in Mplus to combine the estimates using Rubin’s rules (White, Royston, & Wood, 2011). While there is not a well-established technique for addressing missing data in LCA, the approach in the current paper has been used previously (Kretschmer et al., 2014).

Table S2. Proportion of missing data for each study variable.

| **Variable** | Valid (%) | Missing (%) |
| --- | --- | --- |
| **Exposures** |  |  |
| Belief in a higher power | 4158 (99.8%) | 7 (0.2%) |
| Praying when in trouble | 4147 (99.6%) | 18 (0.4%) |
| Attending a place of worship | 4124 (99.0%) | 41 (1.0%) |
| How religious they consider themselves to be | 4131 (99.2%) | 34 (0.8%) |
| How spiritual they consider themselves to be | 4127 (99.1%) | 38 (0.9%) |
| Feel like god has helped them | 4151 (99.7%) | 14 (0.3%) |
| Appeal to god when in trouble | 4147 (99.6%) | 18 (0.4%) |
| Duration of belief | 4069 (97.7%) | 96 (2.3%) |
| Help from religious groups | 3954 (94.9%) | 211 (5.1%) |
| Feels the presence of a higher power | 4125 (99.0%) | 40 (1.0%) |
| Religion lies behind their approach to life | 4120 (98.9%) | 45 (1.1%) |
| Goes to place of worship to make friends | 4120 (98.9%) | 45 (1.1%) |
| Prays for relief and protection | 4107 (98.6%) | 58 (1.4%) |
| **Outcomes** |  |  |
| Wellbeing | 3333 (80.0%) | 832 (20.0%) |
| Depressive symptoms | 3330 (80.0%) | 835 (20.0%) |
| Anxiety | 3360 (80.7%) | 805 (19.3%) |
| Self-harm | 3432 (82.4%) | 733 (17.6%) |
| EPDS depression | 3241 (77.8%) | 924 (22.2%) |
| Self-harm thoughts | 3432 (82.4%) | 733 (17.6%) |
| **Confounds** |  |  |
| Wellbeing | 2988 (71.7%) | 1177 (28.3%) |
| Depressive symptoms | 3026 (72.7%) | 1139 (27.3%) |
| Anxiety | 3041 (73.0%) | 1124 (27.0%) |
| Self-harm | 3108 (74.6%) | 1057 (25.4%) |
| Self-harm thoughts | 3128 (75.1%) | 1037 (24.9%) |
| Income | 2882 (69.2%) | 1283 (30.8%) |
| No educational qualifications | 3211 (77.1%) | 954 (22.9%) |
| Years of education | 2868 (68.9%) | 1297 (31.1%) |
| Social class | 3650 (87.6%) | 515 (12.4%) |
| Maternal belief in higher power | 3341 (80.2%) | 824 (19.8%) |
| Maternal attendance at a place of worship | 3307 (79.4%) | 858 (20.6%) |
| Stressful life events | 2961 (71.1%) | 1204 (28.9%) |
| Relationship quality - offspring reported | 2871 (68.9%) | 1294 (31.1%) |
| Relationship quality - parent reported | 2900 (69.6%) | 1265 (30.4%) |
| Parental monitoring - offspring reported | 2875 (69.0%) | 1290 (31.0%) |
| Parental monitoring - Parent reported | 2644 (63.5%) | 1521 (36.5%) |

**Table S3**. Conditional probabilities for latent class solutions

|  | **Atheist** | **Agnostic** | **Moderately religious** | **Highly religious** |
| --- | --- | --- | --- | --- |
| **Class share:** | **.64** | **.19** | **.13** | **.04** |
| **Belief in god/divine power** |  |  |  |  |
| Yes | .05 | .22 | .93 | .99 |
| Not sure | .25 | .77 | .07 | .01 |
| No | .70 | .01 | .00 | .00 |
| **Pray when in trouble** |  |  |  |  |
| Yes | .01 | .07 | .44 | 1.00 |
| Not sure | .01 | .19 | .19 | .01 |
| No | .99 | .74 | .37 | .00 |
| **Attendance** |  |  |  |  |
| Occasional | .01 | .07 | .17 | .96 |
| Regular | .99 | .93 | .83 | .04 |
| **Importance of religion** |  |  |  |  |
| Very/moderately | .00 | .01 | .20 | .75 |
| Slightly | .02 | .34 | .61 | .16 |
| Not at all | .98 | .66 | .19 | .10 |
| **Importance of spirituality** |  |  |  |  |
| Very/moderately | .05 | .17 | .39 | .89 |
| Slightly | .21 | .42 | .40 | .10 |
| Not at all | .74 | .41 | .21 | .01 |
| **Has God helped them** |  |  |  |  |
| Yes | .00 | .02 | .69 | .94 |
| Not sure | .02 | .77 | .28 | .06 |
| No | .98 | .21 | .03 | .00 |
| **Would they appeal to God** |  |  |  |  |
| Yes | .02 | .23 | .88 | .98 |
| Not sure | .13 | .68 | .10 | .01 |
| No | .86 | .10 | .03 | .00 |
| **Duration of belief** |  |  |  |  |
| More than 5 years | .98 | 1.00 | .97 | .92 |
| Less than 5 years | .02 | .00 | .03 | .08 |
| **Help from religious individuals** |  |  |  |  |
| Yes | .00 | .02 | .04 | .84 |
| No | 1.00 | .98 | .96 | .17 |
| **Feels the presence of divine** |  |  |  |  |
| Definitely true of me | .00 | .01 | .32 | .97 |
| Tends to be true | .01 | .23 | .40 | .03 |
| Unsure | .01 | .19 | .24 | .00 |
| Strongly disagree/not applicable | .98 | .57 | .03 | .00 |
| **Religion lies behind approach to life** |  |  |  |  |
| Definitely true of me | .02 | .04 | .23 | .92 |
| Tends to be true | .02 | .09 | .27 | .06 |
| Unsure | .01 | .12 | .45 | .02 |
| Strongly disagree/not applicable | .95 | .75 | .06 | .00 |
| **Attends church to make friends** |  |  |  |  |
| Definitely true of me | .00 | .02 | .08 | .63 |
| Tends to be true | .00 | .02 | .06 | .02 |
| Unsure | .00 | .00 | .39 | .28 |
| Strongly disagree/not applicable | .99 | .96 | .48 | .07 |
| **Prays for relief and protection** |  |  |  |  |
| Definitely true of me | .00 | .08 | .41 | .52 |
| Tends to be true | .00 | .07 | .17 | .09 |
| Unsure | .00 | .02 | .25 | .38 |
| Strongly disagree/not applicable | 1.00 | .83 | .18 | .02 |

**Table S4**. Results for the complete case sample

| Unadjusted | |  |  | Omnibus p-value | Fully adjusted |  |  | Omnibus p-value |
| --- | --- | --- | --- | --- | --- | --- | --- | --- |
| SMFQ depression | OR | CI | CI | 0.226 | OR | CI | CI | 0.251 |
| Agnostic | 1.62 | 0.92 | 2.84 |  | 1.55 | 0.70 | 3.42 |  |
| Moderately religious | 1.48 | 0.80 | 2.76 |  | 1.33 | 0.57 | 3.08 |  |
| Highly Religious | 0.73 | 0.24 | 2.22 |  | 0.43 | 0.14 | 1.37 |  |
|  |  |  |  |  |  |  |  |  |
| EPDS depression | |  |  | 0.074 |  |  |  | 0.550 |
| Agnostic | 1.56 | 0.90 | 2.72 |  | 1.28 | 0.56 | 2.93 |  |
| Moderately religious | 1.91 | 1.08 | 3.40 |  | 1.67 | 0.74 | 3.73 |  |
| Highly Religious | 1.84 | 0.81 | 4.20 |  | 1.45 | 0.55 | 3.82 |  |
|  |  |  |  |  |  |  |  |  |
| GAD-7 Anxiety | |  |  | 0.087 |  |  |  | 0.085 |
| Agnostic | 1.35 | 0.78 | 2.31 |  | 1.17 | 0.54 | 2.55 |  |
| Moderately religious | 1.91 | 1.10 | 3.31 |  | 2.19 | 1.12 | 4.28 |  |
| Highly Religious | 0.71 | 0.26 | 1.97 |  | 0.56 | 0.18 | 1.73 |  |
|  |  |  |  |  |  |  |  |  |
| Self-harm actions | |  |  | 0.764 |  |  |  | 0.788 |
| Agnostic | 0.94 | 0.31 | 2.83 |  | 0.88 | 0.12 | 6.60 |  |
| Moderately religious | 1.65 | 0.62 | 4.39 |  | 1.88 | 0.52 | 6.77 |  |
| Highly Religious | 1.27 | 0.27 | 5.91 |  | 1.48 | 0.19 | 11.58 |  |
|  |  |  |  |  |  |  |  |  |
| Self-harm thoughts | |  |  | 0.408 |  |  |  | 0.513 |
| Agnostic | 1.15 | 0.66 | 2.01 |  | 1.01 | 0.41 | 2.49 |  |
| Moderately religious | 1.21 | 0.67 | 2.21 |  | 1.33 | 0.58 | 3.06 |  |
| Highly Religious | 0.40 | 0.11 | 1.40 |  | 0.35 | 0.08 | 1.58 |  |
|  |  |  |  |  |  |  |  |  |
| WEMWBS Wellbeing | |  |  | 0.707 |  |  |  | 0.669 |
| Agnostic | 1.32 | 0.79 | 2.22 |  | 1.23 | 0.57 | 2.67 |  |
| Moderately religious | 0.94 | 0.51 | 1.73 |  | 0.72 | 0.31 | 1.63 |  |
| Highly Religious | 0.92 | 0.38 | 2.23 |  | 0.69 | 0.23 | 2.04 |  |

References

Angold, A., & Costello, E. J. (2013). *Short Mood and Feelings Questionnaire* [Data set]. https://doi.org/10.1037/t15197-000

Angulo, M., Rooks, B. T., Gill, M., Goldstein, T., Sakolsky, D., Goldstein, B., … Birmaher, B. (2017). Psychometrics of the screen for adult anxiety related disorders (SCAARED)- A new scale for the assessment of DSM-5 anxiety disorders. *Psychiatry Research*, *253*, 84–90. https://doi.org/10.1016/j.psychres.2017.02.034

Birmaher, B., Brent, D. A., Chiappetta, L., Bridge, J., Monga, S., & Baugher, M. (1999). Psychometric Properties of the Screen for Child Anxiety Related Emotional Disorders (SCARED): A Replication Study. *Journal of the American Academy of Child & Adolescent Psychiatry*, *38*(10), 1230–1236. https://doi.org/10.1097/00004583-199910000-00011

Buuren, S. V., & Groothuis-Oudshoorn, K. (2011). **mice**: Multivariate Imputation by Chained Equations in *R*. *Journal of Statistical Software*, *45*(3). https://doi.org/10.18637/jss.v045.i03

Eyre, O., Bevan Jones, R., Agha, S. S., Wootton, R. E., Thapar, A. K., Stergiakouli, E., … Riglin, L. (2021). Validation of the short Mood and Feelings Questionnaire in young adulthood. *Journal of Affective Disorders*, *294*, 883–888. https://doi.org/10.1016/j.jad.2021.07.090

Kretschmer, T., Hickman, M., Doerner, R., Emond, A., Lewis, G., Macleod, J., … Heron, J. (2014). Outcomes of childhood conduct problem trajectories in early adulthood: Findings from the ALSPAC study. *European Child & Adolescent Psychiatry*, *23*(7), 539–549. https://doi.org/10.1007/s00787-013-0488-5

Kwong, A. S. F., Pearson, R. M., Adams, M. J., Northstone, K., Tilling, K., Smith, D., … Timpson, N. J. (2021). Mental health before and during the COVID-19 pandemic in two longitudinal UK population cohorts. *The British Journal of Psychiatry*, *218*(6), 334–343. https://doi.org/10.1192/bjp.2020.242

Tennant, R., Hiller, L., Fishwick, R., Platt, S., Joseph, S., Weich, S., … Stewart-Brown, S. (2007). The Warwick-Edinburgh Mental Well-being Scale (WEMWBS): Development and UK validation. *Health and Quality of Life Outcomes*, *5*(1), 63. https://doi.org/10.1186/1477-7525-5-63

Thapar, A., & McGuffin, P. (1998). Validity of the shortened Mood and Feelings Questionnaire in a community sample of children and adolescents: A preliminary research note. *Psychiatry Research*, *81*(2), 259–268. https://doi.org/10.1016/S0165-1781(98)00073-0

White, I. R., Royston, P., & Wood, A. M. (2011). Multiple imputation using chained equations: Issues and guidance for practice. *Statistics in Medicine*, *30*(4), 377–399. https://doi.org/10.1002/sim.4067
